# Chromosome-level haplotype-resolved genome assembly for *Takifugu ocellatus* using PacBio and Hi-C technologies

**DOI:** 10.1038/s41597-023-01937-2

**Published:** 2023-01-11

**Authors:** Qingmin Zeng, Zhixiong Zhou, Qian He, Leibin Li, Fei Pu, Mengzhen Yan, Peng Xu

**Affiliations:** 1 Fisheries Research Institute of Fujian, Xiamen, 361000 China; 2 State Key Laboratory of Marine Environmental Science, College of Ocean and Earth Sciences, Xiamen University, Xiamen, 361102 China; 3 Fujian Key Laboratory of Genetics and Breeding of Marine Organisms, College of Ocean and Earth Sciences, Xiamen University, Xiamen, 361102 China

**Keywords:** Genomics, Genome

## Abstract

*Takifugu* species serve as a model system for evolutionary studies due to their compact genomes and diverse phenotypes. The ocellated puffer (*Takifugu ocellatus*), characterized by special colouration, is a scarce anadromous species in the genus *Takifugu*. As an ornamental and tasty fish species, *T. ocellatus* has moderate economic value. However, the available genomic resources for this pufferfish are still limited. Here, a chromosome-level reference genome, as well as two haploid genomes, was constructed by PacBio HiFi long sequencing and Hi-C technologies. The total length of the reference genome was 375.62 Mb with a contig N50 of 11.55 Mb. The assembled sequences were anchored to 22 chromosomes with an integration efficiency of 93.78%. Furthermore, 28,808 protein-coding genes were predicted. The haplotype-resolved reference genome of *T. ocellatus* provides a crucial resource for investigating the explosive speciation of the *Takifugu* genus, such as elucidating evolutionary histories, determining the genetic basis of trait evolution, and supporting future conservation efforts.

## Abstract (summary)

**Table:** 

Measurement(s)	Whole Genome Sequencing • Chromosome assembly by Hi-C data • Whole Transcriptome Sequencing
Technology Type(s)	PacBio Sequel System • Hi-C • Illumina HiSeq. 2500 • Illumina NovaSeq. 6000
Sample Characteristic - Organism	*Takifugu ocellatus*

## Background & Summary

The genus *Takifugu* belongs to the family Tetraodontidae, which inhabits the northwest Pacific Ocean around the coastal area of east Asia^1^. *Takifugu* is composed of approximately 25 species^2^, which are well known for their inflation behaviour and potent neurotoxins. Meanwhile, the group exhibits diverse morphological characteristics and different ecological habits, as well as a compact genome, providing a great model for investigating species radiation. Four high-quality chromosome-level genomes of *T. rubripes*^3^, *T. bimaculatus*^4^, *T. flavidus*^5^, and *T. obscurus*^6^ have been completed in the genus *Takifugu* since the first teleost genome of *T. rubripes* was published in 2002^1^, among which *T. obscurus* and *T. ocellatus* have the capacity for hypotonic adaptation^7^. The ocellated puffer *Takifugu ocellatus* in this study is harboured in China and Vietnam, and is commonly utilized as an ornamental fish species for culture. *T. ocellatus* exhibits saddle-shaped black dots profiled with orange in the dorsal region, in addition to featuring the capacity for euryhaline acclimation, both making it favour aquarium fish. Despite its deadly toxicity, it is also considered a delicacy in East Asia. Therefore, the species has considerable commercial value due to its ornamental value and edibleness. As an anadromous fish, *T. ocellatus* shares the same spawning sites and a similar diet to *T. obscurus*. They acclimate to a broad spectrum of saline water and migrate into freshwater to spawn, while the larvae remain there before emigrating to the seawater. Despite the abovementioned similarities, these two pufferfishes employ different reproductive strategies^7^. In addition, recent phylogenetic analyses in the *Takifugu* genus have shown that they belong to different sister groups, implying that they may be independent of each other in the evolutionary process of adapting to freshwater^8^. Therefore, a high-quality reference genome of *T. ocellatus* is essential to elucidate the speciation process during adaptive radiation, including clarifying the evolutionary histories and adaptation strategies.

In this work, we constructed a chromosome-level genome of *T. ocellatus* by combining PacBio HiFi (high fidelity) reads and Hi-C sequencing data. The genome assembly spanned 375.62 Mb consisting of 163 contigs with a contig N50 length of 11.55 Mb. After chromosome-level anchoring, 22 chromosomes with a total length of 352.28 Mb (93.78% of the draft assembly) were constructed corresponding to the karyotype. Moreover, 66.65 Mb (17.74% of the assembly) of repeat elements, and 28,808 protein-coding genes were annotated. Additionally, two chromosome-level haplotype genome assemblies were also constructed, which would serve as a baseline for future studies on allele-specific expression or conservation genomics. A contiguous and accurate reference genome is essential for basic genetic research and will facilitate evolutionary studies on this euryhaline and anadromous species. In addition, phylogenetic analysis indicated that *T. ocellatus* speciated from the common ancestor of *Takifugu* at approximately 21.4 (mya; 15.3–27.6 mya). We identified 789 gene families with expansion, 1,970 families with contraction, 1,034 rapidly evolving genes, and 767 positively selected genes in *T. ocellatus*. These results will help us to further explore the genetic basis of the freshwater adaptability of *T. ocellatus* and the explosive speciation mechanism of *Takifugu* species.

## Methods

### Sample collection and nucleic acid extraction

Healthy female *T. ocellatus* were collected from Fujian Takifugu Breeding Station in Zhangzhou, Fujian Province, China. Muscle, eye, skin, gill, kidney, liver, intestine, spleen, gonad, heart and stomach were sampled and frozen in liquid nitrogen immediately and then transferred to −80 °C for storage. Genomic DNA (gDNA) of *T. ocellatus* was extracted from enough muscle tissues following the manufacturer’s protocol by an AMPure bead cleanup kit (Beckman Coulter, High Wycombe, UK), while total RNA was extracted from all tissues by a TRIzoL kit and mixed equally for transcriptome sequencing. The quality of gDNA and RNA was detected by 1.5% agarose gel electrophoresis and DNA was quantified by a Qubit fluorometer (Thermo Fisher Scientific, Waltham, MA).

### Library construction and sequencing

According to the manufacturer’s instructions, paired-end libraries for genome surveys with a 350 bp insert size were constructed using gDNA. Then, we sequenced this library with a strategy of 2 × 150 bp on the Illumina HiSeq. 2500 platform and obtained the raw data. For HiFi read generation, high-molecular-weight (HMW) gDNA was sheared to 15 Kbp before preparing a PacBio HiFi library. The genomic library was sequenced in CCS mode on the PacBio Sequel II system at Novogene (Tianjin). Subsequently, HiFi reads were generated from raw subreads using CCS workflow^9^ (v4.2.0) with a recommended setting. Finally, 31.20 Gb of CCS reads were yielded with a mean read length of 16.1 Kb resulted in 84-fold coverage of the *T. ocellatus* genome. The coverage was sufficient for haplotype-resolved assembly according to recommendations^10^. For Hi-C library construction, the *MboI* restriction enzyme was used to digest the cross-linked high molecular weight (HMW) gDNA. After 5′ overhang biotinylated and blunt-end ligation, the DNA was physically sheared into 300–500 bp fragments. Finally, the Hi-C library was sequenced with a strategy of 2 × 150 bp on the Illumina HiSeq. 2500 platform. In addition, 29.16 Gb of paired-end clean reads were generated from the Hi-C library. The RNA-seq library was constructed using the Illumina standard protocol (San Diego, CA, United States) and sequenced on the Illumina HiSeq. 6000 platform. In total, 32.47 Gb of paired-end short clean reads were generated from the RNA-seq library (Table 1).

**Table 1:** Statistics for the sequencing data of the *T. ocellatus* genome.

Library type	Insert Size (bp)	Raw Data (Gb)	Clean Data (Gb)	Average Read Length (bp)	N50 Read Length (bp)	Sequencing Coverage (X)
Illumina	350	53.94	53.67	150	150	145.26
PacBio	15,000	31.2	—	16,053.50	16423	84.44
Hi-C	—	30.89	29.16	150	150	78.92
RNA-Seq	—	32.96	32.47	150	150	87.88
Total	—	148.99	—	—	—	396.5

Note: Genome size estimated by genome survey (369.48 Mb) were used for sequencing coverage calculation.

### Genome survey and assembly

**Fig. 1:**
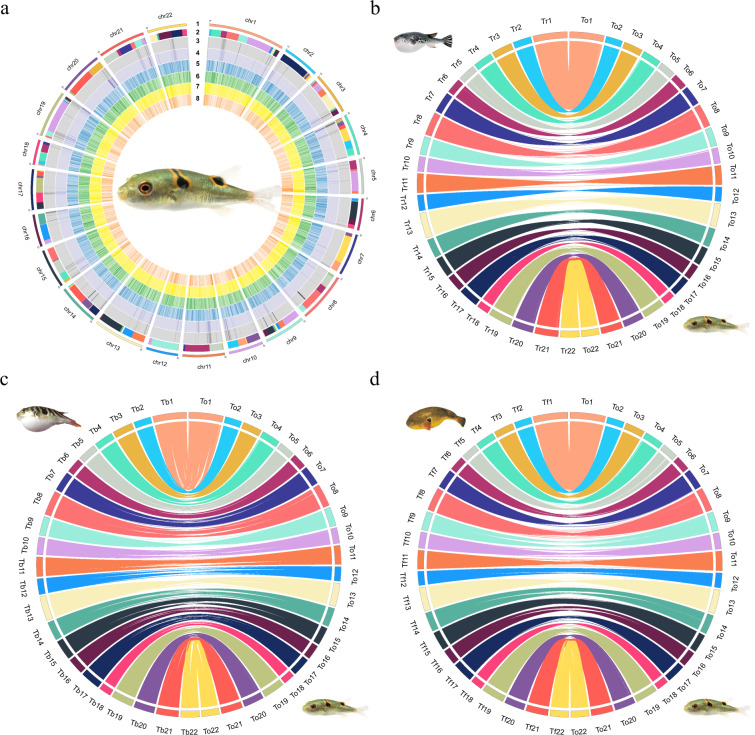
Circos plots of the reference genome of *T. ocellatus* and syntenic relationship with other *Takifugu* species. (**a**): Circos plot of 22 chromosome-level scaffolds, representing annotation results of genes and transposable elements on these scaffolds. The tracks are: (1) 22 chromosome-level scaffold, (2) contigs which comprised the scaffolds (adjacent contigs on a scaffold are painted in different colors, (3) TE abundance of negative strand (grey), (4) TE abundance of positive-strand (purple), (5) gene length of negative strand (blue), (6) gene length of positive strand (green), (7) gene number of negative strand (yellow), (8) gene number of positive-strand (orange); (**b**): Circos diagrams showing *T. ocellatus* chromosome synteny relations with *T. rubripes*; (**c**): Circos diagrams showing *T. ocellatus* chromosome synteny relations with *T. flavidus*; (**d**): Circos diagrams showing *T. ocellatus* chromosome synteny relations with *T. bimaculatus*. Each coloured line in (**b**), (**c**) and (**d**) represents a 1 Kbp fragment match between two species. Chromosome numbers of *T. ocellatus* were re-ordered for better illustration.

Before assembly, the adapter sequences and low-quality reads generated from the Illumina platform were filtered using fastp (v. 0.23.1) software^11^., and the remaining reads were used for subsequent genome survey and assembly. To estimate the major characteristics of the genome, such as genome size, heterozygosity, and repeatability, genome surveys were performed using SOAPec (v. 2.01) and GenomeScope (v. 2.0) software with 17 K-mer frequencies. With a dominant peak depth of 124.92, the estimated genome size of *T. ocellatus* was 369.48 Mb, and the heterozygosity and repetitive sequence content were approximately 0.47 and 27.29%, respectively (Supplementary Table 1 and Supplementary Fig. 1). The estimated genome size is slightly smaller than that of other *Takifugu* species that were assembled by PacBio (previously reported; 373∼404 Mb)^4,6^. Then, the HiFi long reads along with paired-end Hi-C short reads were provided to HiFiasm^12^ (v0.16.1) to generate the monoploid and a pair of haplotype-resolved assembly contig graphs with default parameters. Using the Hi-C integrated algorithm, HiFiasm takes full advantage of phased graphs and long-range information to generate a haplotype-resolved assembly. Finally, three preliminary assemblies, including one monoploid assembly and two haploid assemblies, were yielded, which spanned 375.62 Mb (monoploid), 373.25 Mb (Haploid-1) and 372.15 Mb (Haploid-2), with a contig N50 length of 11.55 Mb, 4.86 Mb and 4.87 Mb, respectively (Table 2). The genome assembly was slightly larger than the estimated genome size of 369.48 Mb (Table 1) because some repeat fragments could be assembled by high-precision CCS reads^13^. Juicer^14^ and 3D-DNA^15^ were implemented to obtain the chromosome-level whole genome assembly for *T. ocellatus*. Afterwards, scaffolds were fine-tuned, and discordant contigs were removed from scaffolds by Juicebox^16^ assembly tools (Table 3; Fig. 1 and Supplementary Fig. 2).

**Table 2:** Statistics of genome assemblies of monoploid and two haploids.

	Monoploid			
	Length		Number	
	Contig (bp)	scaffold (bp)	Contig	scaffold
	Haploid 1			
	Length		Number	
	Contig (bp)	scaffold (bp)	Contig	scaffold
	Haploid 2			
	Length		Number	
	Contig (bp)	scaffold (bp)	Contig	scaffold
Total	3.76E + 08	3.76E + 08	163	329
Max	21489854	29438254	—	—
Number > = 2000 bp	—	—	163	321
N50	11546956	15981353	13	11
N60	9967648	15653316	17	13
N70	6284788	15174500	21	15
N80	4870422	13383635	29	18
N90	2125247	12208387	39	21
Total	3.73E + 08	3.73E + 08	361	419
Max	19092708	29252607	—	—
Number > = 2000 bp	—	—	361	401
N50	4863888	15828131	22	11
N60	4235037	15536097	30	13
N70	2924692	15058794	41	15
N80	1854920	13429000	57	18
N90	793959	12355692	85	21
Total	3.72E + 08	3.72E + 08	264	278
Max	15233066	29304300	—	—
Number > = 2000 bp	—	—	264	267
N50	4867811	16134606	21	10
N60	4203384	15704381	29	13
N70	3030656	15169433	39	15
N80	1866533	13456025	55	18
N90	814732	12383119	86	21

**Table 3:** Statistics of 22 chromosomes of monoploid and two haploids.

	monoploid		haploid 1		haploid 2	
	Length (bp)	Number of Contigs	Length (bp)	Number of Contigs	Length (bp)	Number of Contigs
Chr1	29438254	6	29252607	16	29304300	20
Chr2	14244816	4	14280067	9	13994784	11
Chr3	16307980	3	16640426	10	16772708	11
Chr4	15986684	5	15828131	11	16280894	7
Chr5	13432929	6	13606500	10	13801740	12
Chr6	12208387	6	12507692	9	12606927	7
Chr7	16257593	4	16061500	11	16704851	7
Chr8	19358987	2	19279500	5	19072000	11
Chr9	15981353	5	15843227	15	16113760	7
Chr10	13383635	7	13429000	5	13456025	3
Chr11	16274716	7	15877500	2	16134606	12
Chr12	12374571	3	12355692	15	12383119	6
Chr13	19816107	1	19827542	15	19847000	13
Chr14	15944577	5	15631607	8	16083125	5
Chr15	15178816	1	15058794	12	15169433	6
Chr16	12598642	4	12555500	7	12728500	4
Chr17	15653316	4	15536097	8	15704381	7
Chr18	9796431	8	9993095	5	9980321	7
Chr19	18196925	2	18152500	8	17779869	9
Chr20	17278132	2	17496353	10	17576952	12
Chr21	18405298	4	17932245	1	18227000	12
Chr22	15174500	8	15131885	8	15391000	9

### Genomic repeat annotation

**Fig. 2:**
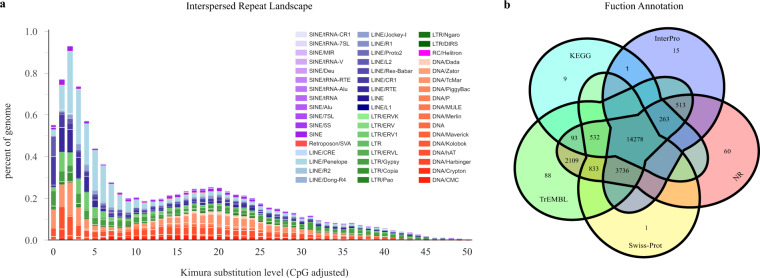
Gene and repeat annotations of the *T. ocellatus* genome. (**a**): Distribution of divergence rate for TEs in the *T. ocellatus* genome. (**b**): Veen diagram of fuctional annotation based on different databases.

Repeat sequences of the *T. ocellatus* genome were identified with both homology-based and *de novo* strategies, following a previously established protocol^17^. First, RepeatModeler was utilized to detect repeats and generate a *de novo* repeat library. Combined with Repbase^18^, an ultimate repeat sequence library was constructed. Finally, RepeatMasker^19^ was employed to forecast repeat elements based on the library. TEclass (v2.1.3) was utilized to further identify unclassified repeats. To summarize the transposable element (TE) annotation results, buildSummary.pl of RepeatMasker was adopted. In addition, the Kimura divergence value of TE was calculated by calcDivergenceFromalign.pl, and TE landscapes were drawn by createRepeatLandscape.pl. Eventually, all repetitive regions were masked in the process of protein-coding gene annotation. By combining Repbase and *de novo* datasets, we obtained a total of 66.65 Mb of consensus and nonredundant repetitive sequences, which occupied more than 17.74% of the monoploid genome (Table 4 & Fig. 2a).

**Table 4:** Classification of repetitive sequences and ncRNAs of the *T. ocellatus* genome.

Repeat type		Denovo + Repbase Length (bp)	Proportion in Genome (%)
ncRNA type		Copy	Proportion in Genome (%)
DNA		23709530	6.31
LINE		22325031	5.94
SINE		876431	0.23
LTR		11011861	2.93
Satellites		12334578	3.28
Simple Repeat		45665	0.01
Unkown		2297329	0.61
Total		66651683	17.74
miRNA		1000	0.028
tRNA		810	0.016
rRNA	18 S	101	0.048
	28 S	489	0.08
	5.8 S	96	0.004
	5 S	887	0.027
	Subtotal	1573	0.159
sRNA	CD-box	112	0.004
	HACA-box	58	0.002
	Splicing	597	0.024
	Subtotal	775	0.031

### Protein-coding gene finding and function annotation

For noncoding RNA (ncRNA) annotation, RNAmmer (v1.2) and tRNAScan (v1.3) were executed for rRNA and tRNA prediction, respectively. Other noncoding RNAs were detected by alignment against the Rfam database. Four types of noncoding RNAs, including 1,000 miRNAs, 810 tRNAs, 1,573 rRNAs and 775 sRNAs, were identified from the *T. ocellatus* genome (Table 4).

Structural annotation of the protein-coding genes was conducted using *ab initio*, homology-based and RNA-seq-based approaches, after all repeat sequences in the *T. ocellatus* genome were soft-masked. For homology-based gene prediction, the protein sequences of *D. rerio*^20^, *O. latipes*^21^, *T. rubripes*^22^, *T. flavidus*^23^ and *T. bimaculatus*^24^ were downloaded from the *European Nucleotide Archive* and provided to GenomeThreader (v.1.7.0)^25^. In addition, the RNAseq clean data were de novo assembled using Trinity software (v.2.10.0). Braker2^26^ was employed to perform *ab initio* gene prediction using the transcripts assembled from RNAseq and known genes of *D. rerio*^20^, *O. latipes*^21^, *T. rubripes*^22^, *T. flavidus*^23^ and *T. bimaculatus*^24^. The optimal parameters were obtained after two rounds of model training. For another gene prediction approach, RNA-seq data were aligned to the *T. ocellatus* genome to assemble the transcriptome via hisat2^27^ and stringtie^28^ (v2.1.4). Then, TransDecoder (v5.5.0) was adopted to predict the open reading frame (ORF) region. Last, a comprehensive gene set was produced by EvidenceModeler and annotated for protein-coding gene structure by PASA (v2.4.1)^29^.

For functional annotation of protein-coding genes, Diamond (v2.0.6) was applied to align protein-coding genes to the NR, TrembBL (http://www.uniprot.org/) and Swiss-Prot (http://www.uniprot.org/) protein databases with E-values < 1*10-5. The annotation of GO and KEGG pathways was performed using InterProScan (v4.8) and KEGG Automatic Annotation Server (KAAS).

### Gene family identification and phylogenetic tree construction

To identify gene families among *T. ocellatus* and other representative species, the protein sequences of *D. rerio* (outgroup), *G. aculeatus*, *H. comes*, *L. litulon*, *M. mola*, *O. latipes*, *T. bimaculatus*, *T. flavidus*, *T. nigroviridis*, *T. palembangensis*, *T. rubripes* and *T. septentrionalis* were downloaded (Supplementary Table 2). Protein sequences shorter than 30 amino acids were filtered out in the above 13 proteome sets and provided to Orthofinder^30^ (v2.5.2) to construct orthologous groups. To reveal the phylogenetic relationships among *T. ocellatus* and 12 other species, single-copy orthologous genes were identified and used for the construction of the phylogenetic tree (Supplementary Table 3). The single-copy orthologues were further aligned using MUSCLE (v3.8.31). Then, RAxML^31^ (v8.2.12) with 1000 bootstrap replicates was executed to generate phylogenetic trees. The divergence time was estimated using MCMCTREE (PAML^32^ package) based on the molecular clock data in the TimeTree^33^ database (fossil time: zebrafish and medaka divided at 230 (million years ago (mya); 180.0–264.0 mya). The expansion and conversion gene families of *T. ocellatus* were identified by CAFÉ (v. 4.2).

### Positive selection and rapidly evolving gene identification

To identify positively selected genes (PSGs) and rapidly evolving genes (REGs) in the *T. ocellatus* genome, the protein sequences of *L. litulon*, *T. bimaculatus*, *T. palembangensis*, *T. rubripes*, and *T. septentrionalis* were downloaded (Supplementary Table 2). We employed the software PRANK-MSA (v140110)^34^ with the parameters gaprate = 0.025 and gapext = 0.75 for coding sequence alignment of each homologous group. To examine the selective constraints on the genes, we estimated the dN/dS ratio (ω) using PAML (v4.4b)^32^. We tested three hypotheses: (1) H0, all branches have the same ω; (2) H1, the branch leading to *T. ocellatus* has a different ω, whereas the other branches have the same ω; and (3) H2, all branches have an independent ω. We used likelihood values and degrees of freedom of the three hypotheses to perform a likelihood-ratio test (LRT). We selected genes whose likelihood values for H1 were significantly larger (adjusted LRT p value of < 0.05) than those for H0 and genes whose likelihood values of H2 were not significantly larger than those of H1. In addition, we also ran branch-site models (model = 2; NSsite = 2) to detect the genes with positively selected sites in *T. ocellatus*. For the null hypothesis, we set ‘fix_omega = 1; omega = 1’, whereas for the alternative hypothesis, we set ‘fix_omega = 0; omega = 1.5’ with the tree ‘(((((*T.bimaculatus*,*T. rubripes*), *T. ocellatus* #1), *T. palembangensis*), *T. septentrionalis*), *L. litulon*)’.

In this study, a high-quality reference genome and two haplotype genomes of *T. ocellatus* were generated, which could contribute to further research on the genetic mechanism of freshwater adaptability and anadromous characteristics. The comparison between the genomes of freshwater-adapted *T. obscurus* and *T. ocellatus* will help us to understand whether there is convergent evolution for freshwater adaptation between these two species. In addition, as the first haplotype-level genome of *Takifugu* species, the *T. ocellatus* genome assembly constructed in this study will facilitate the wide use of *T. obscurus* as a valuable model species to investigate the evolutionary process of adaptive radiation and genetic mechanisms hidden within the compact genome. Combining such information with gene expression data and Hi-C data from different *Takifugu* species, we could deeply explore whether allele-specific gene expression and the 3D structure of the genome would accelerate speciation. Finally, the genome of *T. ocellatus*, as a potential freshwater aquaculture fish, will build a foundation for breeding projects, whose goal is excellent growth traits and freshwater breeding.

## Data Records

The raw sequencing reads of all libraries are available from NCBI via the accession number of SRP407984^35^. The assembled genome is available in the NCBI with the accession number JAPVLW000000000 via the project PRJNA901637^36^. Besides, the assembled genome and sequence annotations are available in the figshare database with the DOI number: 10.6084/m9.figshare.20128412.v1^37^.

## Technical Validation

### Evaluating the completeness of the genome assembly and annotation

To verify the integrity and accuracy of these assemblies, the completeness of the final genome assembly was assessed using Benchmarking Universal Single-Copy Orthologues (BUSCO)^38^ with the lineage database Actinopterygii_odb10. From 3,640 single-copy orthologues, ∼97.5% were fully discovered in the monoploid genome, ∼97.3%, and ∼97.1% were fully found in the Haploid-1 and Haploid-2 genomes (Supplementary Table 4). In addition, the Illumina short reads used for the genome survey were mapped to the genome using BWA^39^ and counted for mapping ratio determination using SAMtools^40^. As a result, the mapping ratios of the three assemblies were 96.19%, 95.65% and 96.03%, and the genome coverages of the three assemblies were 99.84%, 99.86% and 99.84%, respectively (Supplementary Table 5). The consensus quality value (QV) of genomes representing per-base consensus accuracy was estimated by Merqury^41^, and that of all three assemblies exceeded 45 (Supplementary Table 4). In addition, a total of 28,808 nonredundant protein-coding genes were successfully produced by combining *de novo*, homologous searching and transcriptome-assisted predictions. A total of 22,531 genes were successfully functionally annotated (Fig. 2b & Table 5). The number of genes of *T. ocellatus* (27,015) predicted through *de novo* prediction and homolog annotation was slightly greater than that of other species of *Takifugu*, such as *T. bimaclatus* (21,117)^4^ and *T. obscurus* (22,105)^6^, but slightly lower than that of *T. flavidus* (29,416)^5^. Hence, the high integration efficiency, mapping ratio, recognition rate of single-copy orthologues and gene number showed that three assemblies of *T. ocellatus* were of high quality.

**Table 5:** Statistics of gene structure and functional annotation of the *T. ocellatus* genome.

Gene structure Annotation	
Gene fuction Annotation	Number (Percent)
Number of protein-coding gene	28808
Average transcript length (bp)	8860.28
Average exons per gene	14.62
Average exon length (bp)	251.01
Average CDS length (bp)	1665.38
Average intron length (bp)	274.71
Swissprot	19,380 (67.27%)
Nr	22,417 (77.82%)
KEGG	15,177 (52.68%)
InterPro	18,806 (65.28%)
Annotated	22,531 (78.21%)
Unanotated	6,452 (22.40%)

To verify the accuracy of the contig anchoring, three chromosome-level assembled genomes (monoploid genome and two haploid genomes) were first aligned and named after the chromosome number of the published *T. rubripes* genome. Then, the monoploid assembly was aligned to 3 other species in the genus *Takifugu*, including *T. bimaculatus*, *T. flavidus* and *T. rubripes*. Two haploid assemblies were aligned mutually with a unit of 1 Kbp. The 22 chromosomes we identified in the *T. ocellatus* genome aligned exactly against the chromosomes of the other three *Takifugu* species, which suggested a high degree of concordance among them (Fig. 1). The haplotypes also showed strongly reciprocal collinearity (Supplementary Fig. 3).

### Phylogenetic and evolutionary analysis

**Fig. 3:**
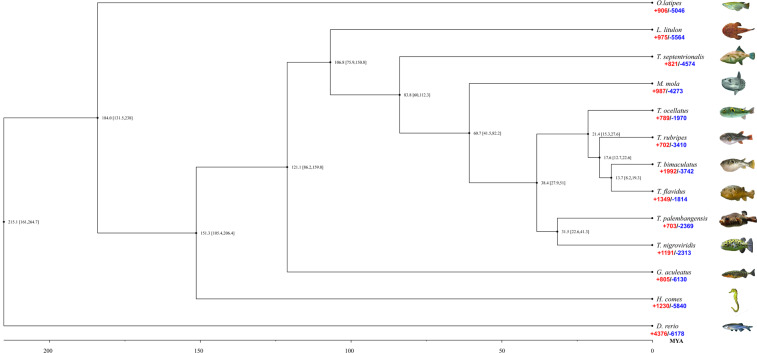
Phylogenetic analysis and divergence time tree of *T. ocellatus* and other representative species. (*D. rerio*, *G. aculeatus*, *H. comes*, *L. litulon*, *M. mola*, *O. latipes*, *T. bimaculatus*, *T. flavidus*, *T. nigroviridis*, *T. palembangensis*, *T. rubripes*, *T. septentrionalis*). The expansion (red) and contraction (blue) gene numbers were listed under the species name.

A total of 21,446 orthologous gene families were identified from the 13 related species (Supplementary Table 3). A total of 2,698 single-copy orthologous gene families in a 1:1:1 manner was identified and used for phylogenetic analysis (Supplementary Table 3). Phylogenetic analysis indicated that *T. ocellatus* speciated from the common ancestor of *Takifugu* at approximately 21.4 (mya; 15.3–27.6 mya) (Fig. 3), which was located at the base of the phylogenetic tree of the *Takifugu* genus, which was consistent with the previous phylogenetic relationship of the *Takifugu* genus based on mitochondrial and whole-genome resequencing^8^. In addition, our results showed that the divergence time between the *Takifugu* genus and the other freshwater Tetraodontidae species was 38.4 mya (27.9–51.0 mya). In addition, the phylogenetic relationship between Tetraodontiformes and other fish was also consistent with previous taxonomic studies^4,5^. Moreover, we uncovered 789 *T. ocellatus* gene families with expansion and 1,970 families with contraction (Fig. 3). GO enrichment analysis showed that the expanded gene families were mainly involved in the extracellular region (GO:0005576), lipid transport (GO:0006869), single-organism transport (GO:0044765) and growth factor activity (GO:0008083) (Supplementary Fig. 4 and Supplementary Table 6). On the other hand, the contracted gene families were mainly involved in xenobiotic transporter activity (GO:0042910), transmembrane transport (GO:0022857), secondary active transmembrane transporter activity (GO:0015291), and hydrolase activity (GO:0016787) (Supplementary Fig. 4 & Supplementary Table 7).

**Fig. 4:**
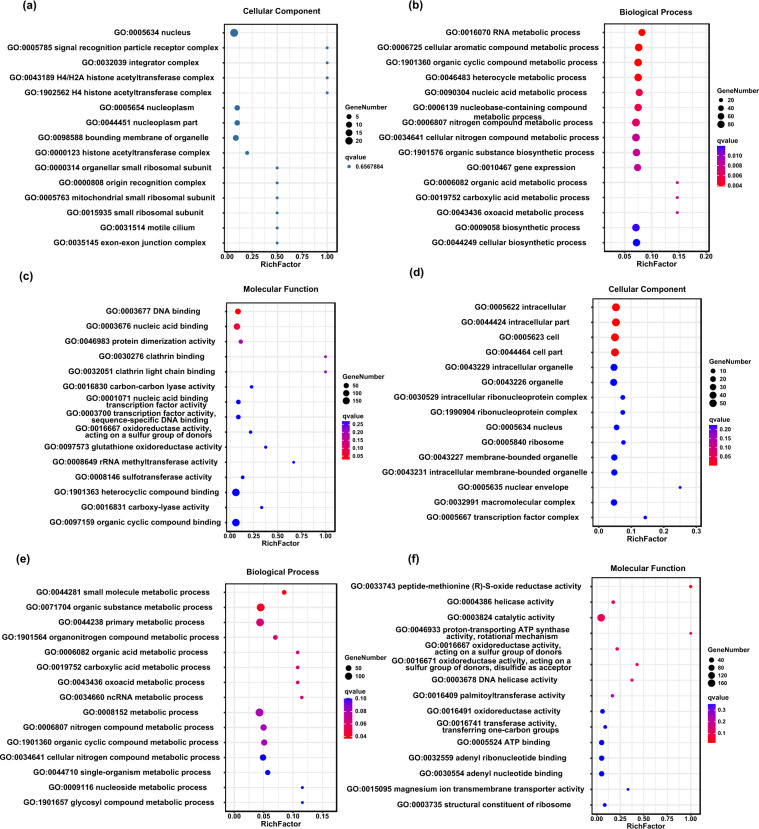
The bubble diagram of GO enrichment of positive selection and rapidly evolving genes in *T. ocellatus*; (**a**–**c**): GO enrichment of the positive selection; (**d**–**f**): GO enrichment of the rapidly evolving genes.

We identified 1,034 rapidly evolving genes with significant false discovery rate (FDR)-corrected p values (<0.05) in *T. ocellatus* (Supplementary Table 8). GO enrichment analysis showed that the REGs were mainly involved in RNA metabolic process (GO:0016070), cellular aromatic compound metabolic process (GO:0006725), nitrogen compound metabolic process (GO:0006807), and gene expression (GO:0010467) (Fig. 4 and Supplementary Table 9). In addition, using an FDR-corrected LRT p-value (adjusted LRT p value) cut-off of 0.05, we identified 767 PSGs in *T. ocellatus* (Supplementary Table 10). GO enrichment analysis showed that the REGs were mainly involved in intracellular (GO:0005622), small molecule metabolic process (GO:0044281), nitrogen compound metabolic process (GO:0006807), and oxoacid metabolic process (GO:0043436) (Fig. 4 & Supplementary Table 11).

For marine fishes, K^+^/Cl^−^ cotransporters (KCCs) play important roles in epithelial ion transport and osmotic homeostasis^6^. In another freshwater-adapted pufferfish, *T. obscurus*, “potassium:chloride symporter activity (GO0015379)” was observed as an expanded gene family, which may be associated with the unique anadromous characteristics of *T. obscurus*^6^. For *T. ocellatus*, we identified several REGs that participated in the osmotic pressure regulation of K+/Cl−, such as potassium channel subfamily K member 3 (*kcnk3*), potassium channel subfamily K member 1 (*kcnk1*) and potassium channel subfamily K member 10 (*kcnk10*) (Supplementary Table 8). In addition, we identified several PSGs that were also involved in K+/Cl- transport, such as potassium channel subfamily K member 5 (*kcnk5*), solute carrier family 12 member 3 (*slc12a3*), and solute carrier family 26 member 6 (*slc26a6*) (Supplementary Table 10). These candidate genes may enhance the osmotic pressure regulation ability so that *T. ocellatus* can adapt to the freshwater environment.

## Supplementary information

Supplymentary Figure

Supplymentary Table

## Data Availability

Genome annotation: (1) RepeatMasker: parameters: -e ncbi -a -nolow -no_is -norna (2) TE-class: parameters: all parameters were set as default (3) Braker2: parameters: all parameters were set as default (4) PASA:–ALIGNERS blat (5) EvidenceModeler: parameters: all parameters were set as default Genome assembly: (1) CCS: parameters: all parameters were set as default (2) HiFiasm: parameters: hifiasm -u -o genome.asm–h1 R1.fq.gz–h2 R2.fq.gz ccs.fa.gz Gene family identification and phylogenetic analysis: (1) RAxML: parameters: -f a -m PROTGAMMAAUTO (2) MCMCTREE: parameters: all parameters were set as default The parameters of other not mentioned analysis modules were used as default parameters. The other custom codes used in this analysis were mentioned in the methods sections.
