# Supplementary material for: Chromosome-level haplotype-resolved genome assembly for Takifugu ocellatus using PacBio and Hi-C technologies
Source: Sci Data. 2023 Jan 11;10:22. doi: 10.1038/s41597-023-01937-2 (PMC9834249; doi:10.1038/s41597-023-01937-2)

Contents

Fig 1. Distribution profiles of 17-mer analysis of Illumina reads. 2
Fig 2. The Hi-C interactive heatmap of three assemblies. 3
Figure S3. Circos diagram showing collinearity between two haploids' scaffoldings. 4
Figure S4. The bubble diagram of GO enrichment of expansion and contraction gene families in *T. ocellatus* 5

Fig 1. Distribution profiles of 17-mer analysis of Illumina reads.

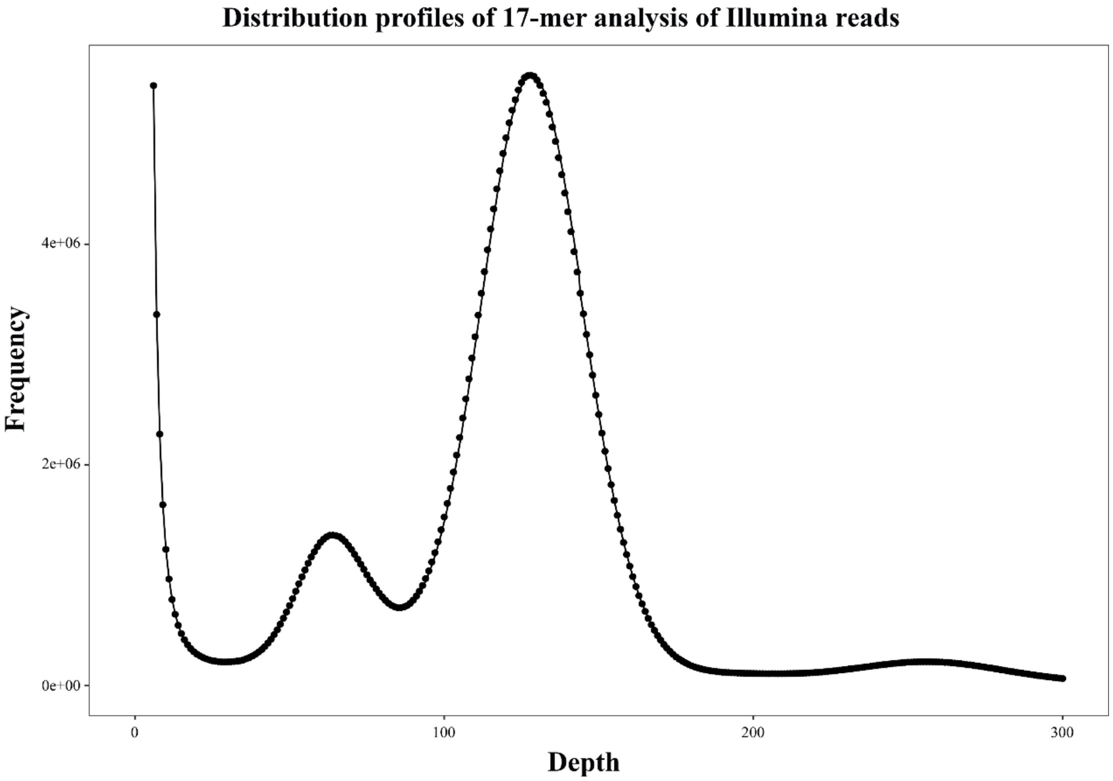

Fig 2. The Hi-C interactive heatmap of three assemblies. (A) Monoploid genome; (B) Haploid-1 genome; (C) Haploid-2 genome.

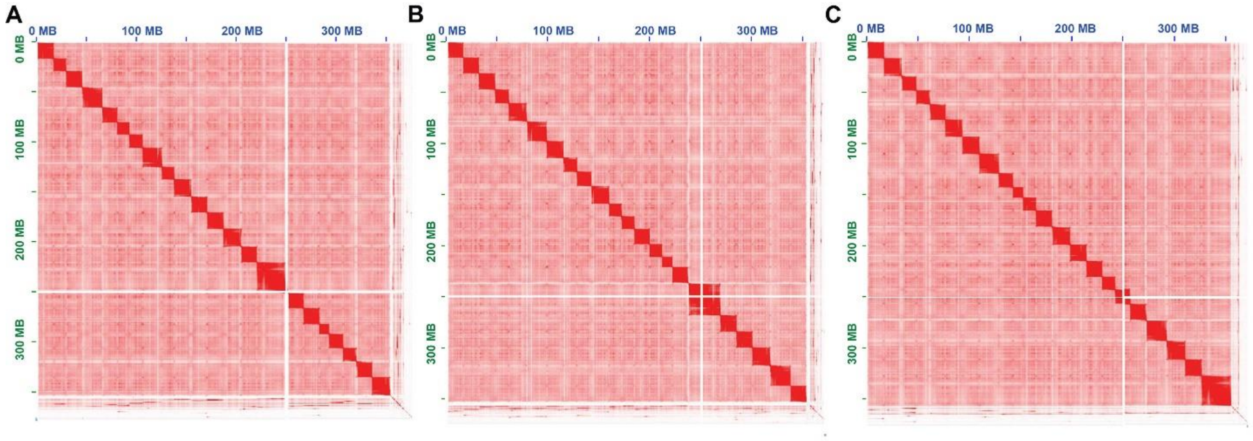

Figure S3. Circos diagram showing collinearity between two haploids' scaffoldings. T1: Haploid-1 genome; T2: Haploid-2 genome; the link line between T1 and T2 represent the strong reciprocal collinearity based on the sliding window of 1 Kbp.

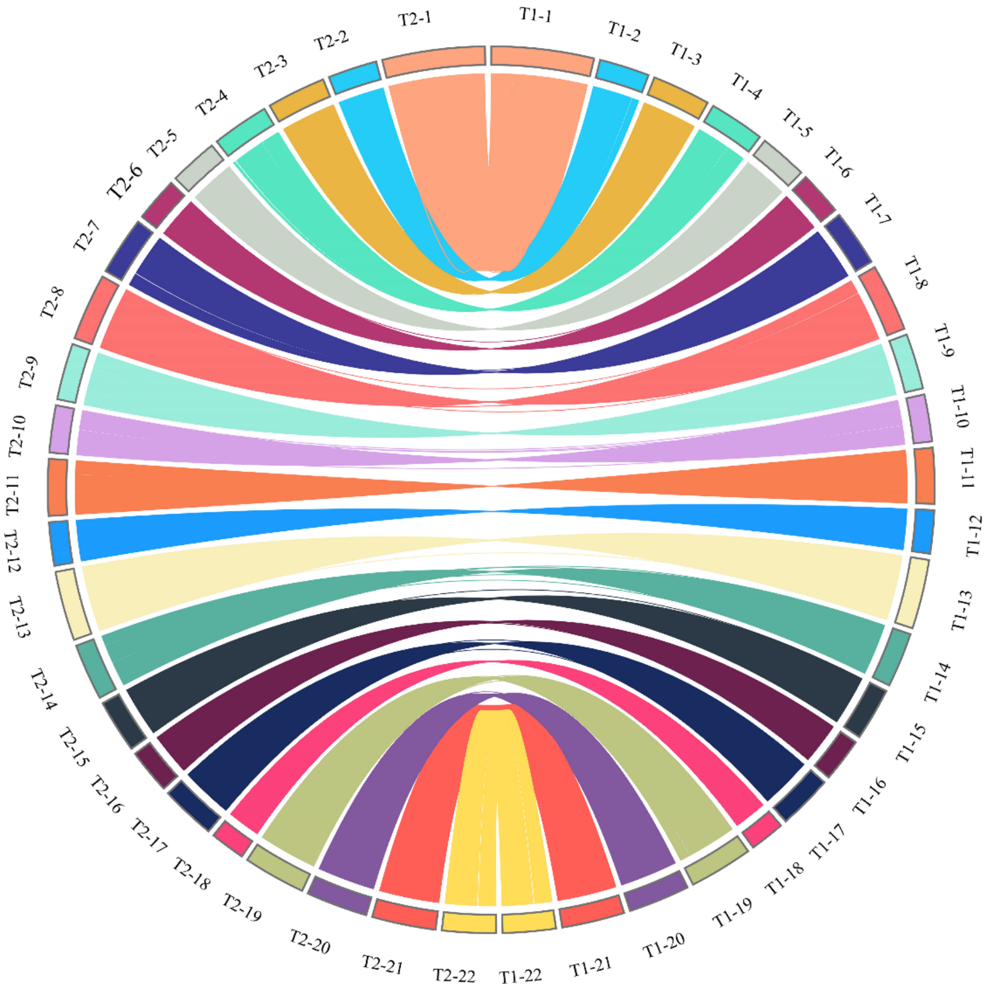

Figure S4. The bubble diagram of GO enrichment of expansion and contraction gene families in T. ocellatus; (A),(B),(C): GO enrichment of the expansion gene families; (D),(E),(F): GO enrichment of the contraction gene families.

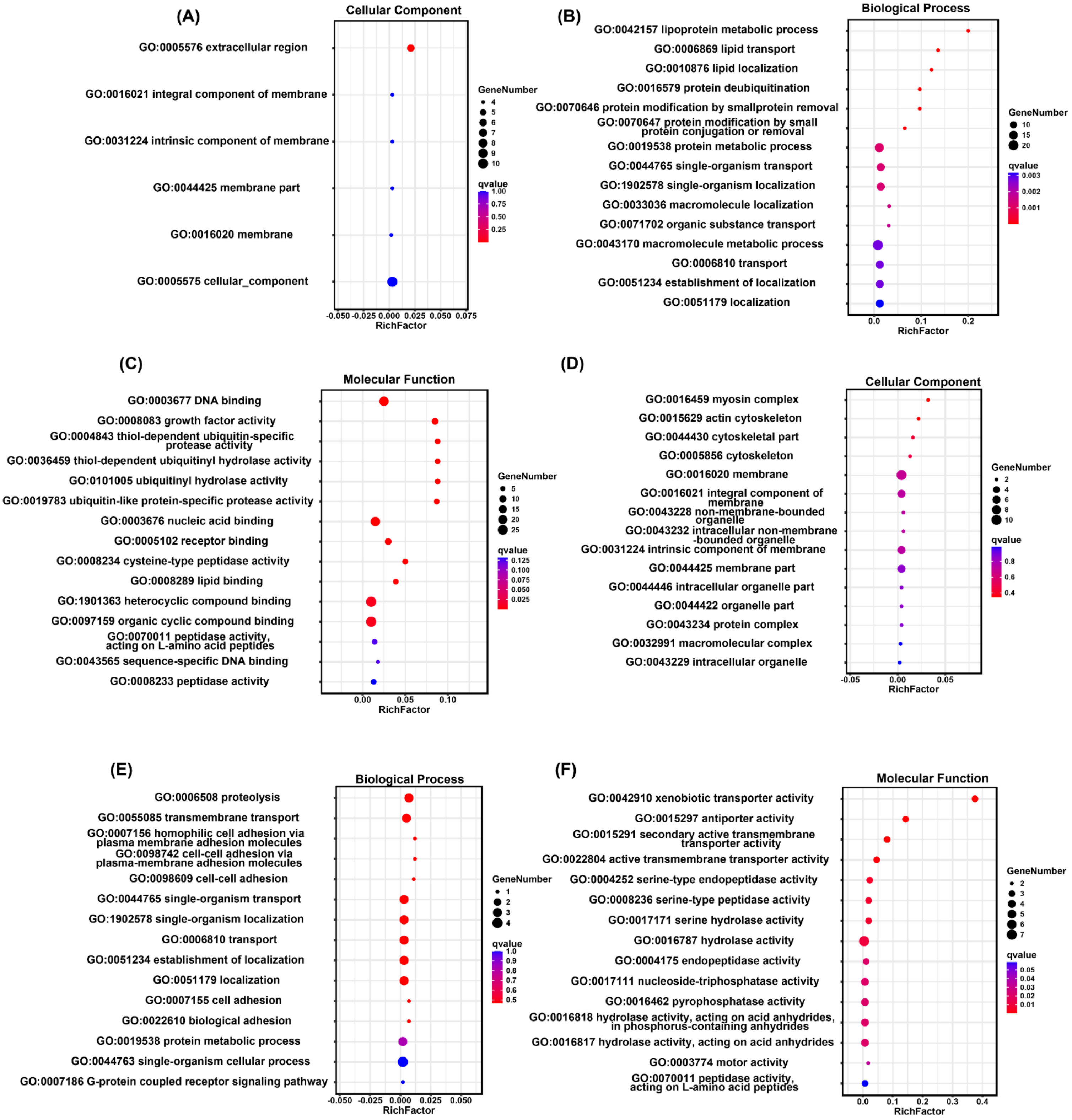

Supplement: Supplementary file 1 — Supplymentary Figure [file 41597_2023_1937_MOESM1_ESM.pdf]
